# Local control of the host immune response performed with mesenchymal stem cells: perspectives for functional intracerebral xenotransplantation

**DOI:** 10.1111/jcmm.12414

**Published:** 2014-10-14

**Authors:** Xavier Lévêque, Elodie Mathieux, Véronique Nerrière-Daguin, Reynald Thinard, Laetitia Kermarrec, Tony Durand, Thomas Haudebourg, Bernard Vanhove, Laurent Lescaudron, Isabelle Neveu, Philippe Naveilhan

**Affiliations:** a INSERM, UMR 1064Nantes, France; b CHU de Nantes, Institut de Transplantation et de Recherche en Transplantation, ITERTNantes, France; c Faculté de Médecine, Université de Nantes, LUNAM UniversitéNantes, France; d INSERM, UMR 913Nantes, France; e INSERM, UMR 791Nantes, France

**Keywords:** immunosuppression, rejection, mesenchymal stem cells, T cells, Parkinson, brain transplantation, survival, restorative strategies

## Abstract

Foetal pig neuroblasts are interesting candidates as a cell source for transplantation, but xenotransplantation in the brain requires the development of adapted immunosuppressive treatments. As systemic administration of high doses of cyclosporine A has side effects and does not protect xenotransplants forever, we focused our work on local control of the host immune responses. We studied the advantage of cotransplanting syngenic mesenchymal stem cells (MSC) with porcine neuroblasts (pNb) in immunocompetent rat striata. Two groups of animals were transplanted, either with pNb alone or with both MSC and pNb. At day 63, no porcine neurons were detected in the striata that received only pNb, while four of six rats transplanted with both pNb and MSC exhibited healthy porcine neurons. Interestingly, 50% of the cotransplanted rats displayed healthy grafts with pNF70+ and TH+ neurons at 120 days post-transplantation. qPCR analyses revealed a general dwindling of pro- and anti-inflammatory cytokines in the striata that received the cotransplants. Motor recovery was also observed following the transplantation of pNb and MSC in a rat model of Parkinson's disease. Taken together, the present data indicate that the immunosuppressive properties of MSC are of great interest for the long-term survival of xenogeneic neurons in the brain.

## Introduction

Transplantation is a promising approach for nervous system repair in neurodegenerative disorders. Clinical studies have shown beneficial effects following the transplantation of embryonic ventral mesencephalic tissue into the brains of Parkinson's disease patients, but the absence of effects or the apparition of dyskinesia in some patients has underlined the need for technical improvements. These include a better control of the host immune response, as alloimmunization to donor antigens has been observed following the transplantation of foetal neural cells into the brains of patients affected by Huntington's disease [1]. Alternative sources of transplantable cells have also to be found as the use of human foetal tissues raises ethical and logistical problems. In this perspective, we have been studying the possibility of using porcine embryonic ventral mesencephalic tissue [2–4]. Porcine foetal neurons have been shown to restore behavioural function in animal models of Parkinson's disease [5,6] and some clinical trials have already been performed [7–9]. However, despite the particular immunological status of the brain, neuronal xenografts become the target of a host immune response characterized by a massive infiltration of dendritic and T cells with a Th1 profile [3,4]. Treatment with minocycline [10] or cyclosporine A significantly delays the rejection without inhibiting it. Interestingly, mesenchymal stem cells (MSC) showed spontaneous long-term survival following their transplantation into the brain of a xenogeneic host [11,12]. This long-term survival is due not only to the low immunogenicity of MSC but also to their active immunosuppressive activity. Indeed, MSC can inhibit the maturation of dendritic cells and proliferation of T- and B cells [13,14]. Interestingly, transplantation of MSC in animal models of stroke or multiple sclerosis revealed that these cells may have beneficial effects through their immune and trophic properties [15]. Consequently, we decided to take advantage of these properties to develop a local immunosuppressive strategy in the brain. Our results showed that MSC protect xenogeneic neural cells from cell rejection without affecting their functional properties.

## Materials and methods

### Animals

Porcine embryos were obtained from domestic Large White pigs 28 days after insemination (G28; Institut National de la Recherche Agronomique, INRA, Nouzilly, France). Lewis 1A and Sprague–Dawley wild-type rats were purchased from Janvier CERJ (Le Genest saint Isle, France) and Charles River (L'Arbresle, France). The experiments were performed according to the French guidelines for the care and use of laboratory animals.

### Culture of rat MSC

Mesenchymal stem cells were obtained from the bone marrow of adult Lewis 1A rats as previously described [12,16,17]. The ends of femurs and tibias were cut, and bone marrow cells (BMC) were aspirated with a 25-gauge needle. To remove all the BMC, the bone canal was flushed with αMEM medium (Sigma-Aldrich, Saint-Quentin Fallavier, France) supplemented with 20% foetal calf serum (FCS; Gibco, Life technologiies ltd, Asint-Aubin, France), 100 U/ml penicillin (Sigma-Aldrich) and 100 μg/ml streptomycin (Sigma-Aldrich). MSC were selected by an overnight incubation of the BMC onto uncoated plastic dishes. Plastic-adherent cells were then plated at a density of 8 × 10^3^ cells/cm^2^ and split at 80% confluency. Full characterization of the cells was previously described [12,17] and current analyses by flow cytometry indicate that over 95% of the cells were consistently positive for CD90 and negative for CD45. MSC were used at passage 4.

### Preparation of porcine neuroblasts

Foetuses at G28 were collected in Hank's balanced salt solution (GibcoBRL) after hysterectomy. Ventral mesencephalons were dissected under aseptic conditions and conserved in a hibernation medium at 4°C for up to 3 days. Just before transplantation, porcine neuroblasts (pNb) were prepared by mechanical and enzymatic dissociation as previously described [2] and the cell suspension was adjusted to a concentration of ∼2 × 10^5^ cells/μl.

### Cell transplantation and surgical procedures

The transplantation was performed on male Lewis 1A rats weighing 250 g. Anaesthesia was performed with an intramuscular injection of 2% Rompun and 50 mg/ml ketamine (1 ml/kg) (PanPharma, Fougères, France). Anaesthetized rats were placed in a stereotaxic frame (Stoelting, Wood Dale, IL, USA). An amount of 4 × 10^5^ pNb, 1 × 10^5^ rat MSC or a mix of 3 × 10^5^ pNb/1 × 10^5^ rMSC was transplanted unilaterally into the striatum (in mm relative to bregma and surface of the dura mater): anterior: +0.7, lateral: −2.8, ventral: −6 and −5.6, incisor bar: −3.3. Injections were performed with a Hamilton syringe placed on an automated microinjector (Phymed, Paris, France) over a time period of 1 min. After a 4-min. delay period, the needle was gently removed, the piece of skull replaced and the skin sutured.

At 28, 35, 42, 63 and 120 days post-transplantation, animals were anaesthetized with an intramuscular injection of 2% Rompun/50 mg/ml ketamine (1 ml/kg) and transcardially perfused with 150 ml 0.9% NaCl, followed by 250 ml cold 4% paraformaldehyde in PBS. The brain was then removed from the skull and cryoprotected in two successive solutions of 15% (24 hrs) and 30% (48 hrs) sucrose in PBS at 4°C. The brains were then frozen in isopentane and stored at −80°C. For the molecular analysis, grafted striata were collected from unperfused animals and directly frozen in liquid nitrogen.

### 6-hydroxydopamine lesion

Lesion of nigral dopaminergic neurons was carried out by injecting, unilaterally, the 6-hydroxydopamine (6-OHDA) in the medial forebrain bundle. Briefly, rats were anaesthetized with an intramuscular injection of Rompun/ketamine (1 ml/kg) and placed in a stereotaxic frame (Stoelting). 6-OHDA (3 μg/μl) was dissolved in 0.9% saline/0.1% ascorbic acid, and 4 μl were unilaterally injected (0.33 μl/min) at the following coordinates (in mm relative to bregma and surface of the dura mater): anterior: −4.4; lateral: −1.2; ventral: −7.8; tooth set bar: −2.3. Two weeks after the lesion, the animals were injected with the DA agonist, apomorphine (0.05 mg/kg, s.c.). Only the animals scoring more than 100 contralateral rotations during a 45-min. period were selected for cell transplantation, as this score corresponds to ∼90% of dopaminergic loss on the side ipsilateral to the lesion ( Fig. 5A). Four experimental groups were then constituted: (*i*) nontransplanted (*n* = 8), or transplanted (*ii*) with pig neuroblasts (*n* = 8), (*iii*) LEW 1A MSC (*n* = 8), (*iv*) MSC plus pig neuroblasts (*n* = 13). Cell transplantation was performed 1 month after the 6-OHDA lesion.

### Cylinder tests

Functional recovery of 6-OHDA-lesioned rats was followed by analysis of forelimb-use asymmetry every 15 days. Rats were placed in a glass cylinder and the number of wall contacts executed independently with the left and right forepaw was counted for each rat (cylinder test) [18]. At least 20 contacts were counted for each animal. Left (contralateral) paw use was expressed as a percentage of the total number of wall contacts, knowing that an unlesioned animal achieves a score of 50%.

### Total RNA preparation and qPCR analyses

To extract total RNA, cells were disrupted in 1 ml TRIzol® (Invitrogen, Carlsbad, CA, USA) and homogenized using a syringe and needle for cells or using an ULTRA-TURRAX T25 (IKA, Staufen, Germany) for tissues according to the manufacturer's specifications.

Potential genomic DNA contamination was removed by treatment with Turbo™ DNase (Ambion Inc., Austin, TX, USA). RNA was quantified using an ND-1000 UV-Vis spectrophotometer (Nanodrop Technologies, Wilmington, DE, USA) and RNA integrity was controlled on an agarose gel. cDNA was synthesized from 5 μg total RNA using the Moloney Murine Leukemia Virus reverse-transcriptase kit (Invitrogen) and diluted to a final concentration of 100 ng cDNA/μl.

Analyses of transcripts were performed with a GenAmp 7700 sequence detection system (Applied Biosystems, Life technologies ltd, Saint-Aubin, France) using SYBR Green PCR core reagents (AB). Oligonucleotide sequences are indicated in Table 1. To minimize the amplification of porcine mRNA, primers were aligned against porcine mRNA sequences and selected as follows: at least, one of the two primers matched on less than nine nucleotides and/or the 3′ end was free if the homology was greater than 50%. These conditions were not satisfied for Glial Fibrillary Acidic Protein (GFAP) and transforming growth factor-beta (TGF-β). The PCR method and the 2^−ΔΔCt^ quantification method, after normalization to HPRT values, have been described previously. The mRNA expression level is defined as the fold change in mRNA levels in a given sample relative to levels in a calibrator (CB). The calibrator is the 1× expression of each gene. The mRNA expression level is calculated as follows: mRNA expression level = 2^−ΔΔCt^ where ΔΔCt = (Ct_Target_ − Ct_HPRT_)_sample_ − (Ct_Target_ − Ct_HPRT_)_CB_. Specific amplifications were checked by amplicon melting curves.

**Table 1:** Primers used in this study

Primers	Sequence	Rattus norvegicus accession
rHPRT	Up CCTTGGTCAAGCAGTACAGCC	NM_012583
	Lo TTCGCTGATGACACAAACATGA	
rCb	Up GTGAATGGGAAGGAGATCCG	AY228549.1
	Lo CACTGATGTTCTGTGTGACAGGTT	
CD11b	Up TTGGAGTTGCCTGTGAAGTACG	NM_012711.1
	Lo TGCGACAGACACTTGAGAGGTT	
rGFAP	Up CTAGCCCTGGACATCGAGATC	NM_017009.2
	Lo TCCTGCTTCGACTCCTTAAT GA	
MCP1	Up CCACTCACCTGCTGCTACTCAT	NM_031530.1
	Lo TTCTGATCTCACTTGGTTCTGGTC	
rRANTES	Up GCATCCCTCACCGTCATCCT	NM_031116.3
	Lo TAGCTCATCTCCAAATAGTTGAT	
IFN-γ	Up TGGATGCTATGGAAGGAAAGA	NM_138880.2
	Lo GATTCTGGTGACAGCTGGTG	
rIL6	Up GCAAGAGACTTCCAGCCAGTT	NM_012589.2
	Lo CATCATCGCTGTTCATACAATCA	
rIL10	Up TGCTATGTTGCCTGCTCTTACTG	NM_012854.2
	Lo TCAAATGCTCCTTGATTTCTGG	
rTGF-β	Up CTCAACACCTGCACAGCTCC	NM_021578.2
	Lo ACGATCATGTTGGACAACTGCT	
rHO-1	Up CCACAGCTCGACAGCATGTC	NM_012580.2
	Lo GTTTCGCTCT ATCTCCTCTT CCA	
iNOS	Up GACCAAACTGTGTGCCTGGA	NM_012611
	Lo TACTCTGAGGGCTGACACAAGG	

### Immunohistochemical analyses

The frozen brains were sliced coronally into 16 μm sections using a cryostat (Leica, Nanterre, France). Parallel series of nine slides, each containing three brain sections, were prepared. The slides were stored at −80°C until further handling. After neutralization of endogenous peroxidase with 0.3% H_2_O_2_ in PBS (Sigma-Aldrich) for 10 min., the slides were incubated for 45 min. in 10% normal goat serum (Sigma-Aldrich) diluted in PBS-4% bovine serum albumin (BSA, Sigma-Aldrich) before an incubation with primary antibodies, as presented in Table 2, overnight at 4°C. After washing, slides were incubated for 1 hr at room temperature with biotinylated antimouse or anti-rabbit Abs (1:500 in PBS-4% BSA). Sections were then washed three times and exposed to avidin-biotinylated-peroxidase complex for 1 hr and revealed with the Vector very intense purple substrate or 3,3-diaminobenzidine (Vector, Laboratories, Burlingame, CA, USA; ABC kit). After several washes in distilled water, slides were dehydrated and mounted using Eukitt (Labonord, Villeneuve d'Ascq, France).

**Table 2:** Antibodies used in this study

Specificity	Host	Clone	Dilution	Target cells	Origin
CD11b	Mouse	Ox42	Supernatant	Macrophages, microglial cells	1
Porcine NF70	Mouse	DP5	10 μg/ml	Porcine neurons	2
T-cell receptor α-β chain	Mouse	R73	Supernatant	T cells	1
TH	Rabbit	Polyclonal	0.1 μg/ml	Dopaminergic neurons	3
Mouse IgG (FITC)	Goat	Polyclonal	1.5 μg/ml		4
Mouse IgG (Biot)	Horse	Polyclonal	1 μg/ml		4
Rabbit IgG (FITC)	Donkey	Polyclonal	3 μg/ml		4
Rabbit IgG (Biot)	Goat	Polyclonal	2.2 μg/ml		4

1, European Collection of Cell Culture, Salisbury, UK; 2, Université Paris VII, Paris, F; 3, PelFreeze, Rogers, AR, USA; 4, Jackson Immunoresearch, Cambridgeshire, UK.

Observations were performed with an Axioskop 2 plus microscope (Zeiss, Marly le Roi, France). Images were acquired using a digital camera (AxioCam HRC, Zeiss) driven by AxioVision Release 4.2 software.

### Histological classification of the graft

Brain sections containing the graft were identified by staining one slide of each series with cresyl violet (CV). In the absence of an identifiable graft because of rejection, the needle track or scar was used to localize the injecting point. A minimum of three sections from the graft centre were stained with pNF70 and R73 antibodies to detect porcine neurons and T lymphocytes respectively. The graft status was assessed by two independent readers on blind-coded slides and classified using the following criteria. Healthy grafts: no sign of degeneration (CV^+^) and/or a clear expression of the porcine NF (NF70^+^), no (R73^−^) or rare (occasionally observed in one or more sections, but always in a much lower number than 10 cells per brain section, R73+/−) T cells inside the graft. Rejecting graft: clear expression of porcine NF (NF70^+^), very large number of T cells infiltrating the graft and/or the grafted striatum (R73^+^). Rejected: no porcine NF (NF70^−^), a very large number of R73^+^ cells infiltrating the graft and/or the grafted striatum (R73^+^). Scar: no porcine NF, no T cells. This classification was chosen as it encompassed the five situations observed after the transplantation of pNB into the rat striatum [4,10,19].

### Efficiency of the 6-OHDA lesion

The efficiency of 6-OHDA lesion was controlled by analysing the percentage of dopaminergic loss in the substantia nigra compacta. The number of TH-positive cells was counted at three different levels of the SNc using, as a reference point, the exit of the third nerve (200 μm rostral and caudal to this site). Cell counting was performed with the Mercator stereology software (Explora nova, La Rochelle, France). The percentage of loss was expressed as the number of TH^+^ cells on the unlesioned side minus the number of TH^+^ cells on the lesioned side, divided by the number TH^+^ cells at the unlesioned side, and multiplied by 100. The results were analysed by two-way anova.

## Results

### MSC promote the long-term survival of porcine mesencephalic neuroblasts following their implantation into the striatum of immunocompetent rats

**Fig. 1:**
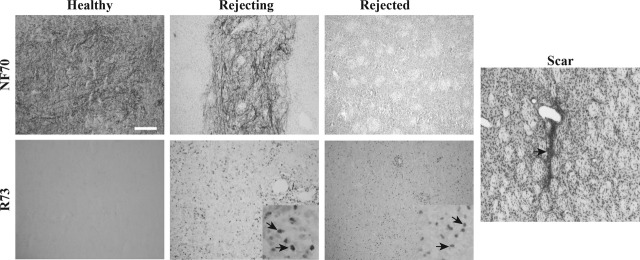
Histological assessment of pNb graft status using pNF70 and R73 antibodies. Healthy grafts exhibit no signs of degeneration, a clear NF70 immunostaining and no (−) or few (+/−) R73 cells (NF70^+^/R73^−^, Healthy). Rejecting grafts are invaded by numerous T cells (enlargement in the insert), but porcine neurons are still present and express pNF70 (NF70^+^R73^+^, Rejecting). Rejected grafts are characterized by the disappearance of pNF70^+^ cells, whereas R73^+^ cells are still observed (enlargement in the insert) (NF70^−^R73^+^). When the rejection process is complete, the T cells have totally disappeared and the graft has become a scar (Scar).

The immunosuppressive and neurotrophic activities of the MSC derived from the bone marrow of Lewis 1A rats prompted us to test their efficiencies to promote the survival of intracerebral xenotransplants. For this purpose, pNb were transplanted into the striata of Lewis 1A rats, with or without Lewis 1A rMSC and the experiments were designed to use the same total number of transplanted cells in all conditions. Survival of the graft was analysed at 28, 35, 42, 63 and 120 days post-transplantation, with a minimum of five rats for each time-point. The presence of porcine neurons in the rat striatum was controlled for using an antibody that specifically recognizes the porcine neurofilament 70 kD subunit (NF70^+^). Infiltration of the graft by T lymphocytes was investigated using R73, an antibody directed against the T-cell receptor. As illustrated in Figure 1, a combination of these two labellings provides a good evaluation of the graft status. At early stages, such as D28, pNb grafts were usually homogeneous with numerous NF70^+^ cells and no T cells (NF70^+^/R73^−^, healthy graft). At intermediate stages, such as D35, pNb grafts were often heterogeneous. Porcine neurons were still present, but the graft was clearly infiltrated by R73^+^ cells (NF70^+^/R73^+^, rejecting graft). At later stages, such as D42, NF70-positive labelling was no longer visible, but R73^+^ cells were still present (NF70^−^/R73^+^, rejected graft). As indicated in Table 3, variations in this timing existed between animals, but the rejection process in Lewis 1A rats transplanted with pNb was systematically completed at D63. At this time, only a scar was observed (Scar). The presence of MSC significantly impaired this rejection process. Indeed, all the pNb/MSC grafts looked healthy at D35 and 50% of the rats transplanted with both pNb and rMSC exhibited healthy grafts at D120 (Table 3).

**Table 3:** Graft survival in rats grafted with pNb or pNb+MSC

	D28		D35		D42		D63		D120
MSC	−	+	−	+	−	+	−	+	+
Number of rats	6	5	6	6	6	5	6	6	6
NF70orCV^+^/R7.3^−^	4	5	1	6	2	1	0	2	3
NF70^+^/R7.3^+/−^	1	0	3	0	0	2	0	1	0
NF70^+^/R7.3^+^	1	0	1	0	2	0	0	1	0
NF70^−^/R7.3^+^	0	0	1	0	2	1	0	1	0
Scar	0	0	0	0	0	1	6	1	3

The graft status was assessed using cresyl violet, NF70 and R73 staining. CV^+^/R73^−^ and NF70^+^/R73^−^ are considered as healthy graft with no (−) or low (+/−) amount of R73 T cells, no sign of degeneration (CV^+^) and/or a clear expression of the porcine neurofilament in neurons (NF70^+^). NF70^+^R73^+^ and NF70^−^R73^+^ grafts are considered as rejecting and rejected graft respectively. Scar corresponds to the stage of almost complete disappearance of the graft. R73^+/−^ corresponds to a brain with 1–10 T cells per section.

### Characterization of pNb-MSC cografts

**Fig. 2:**
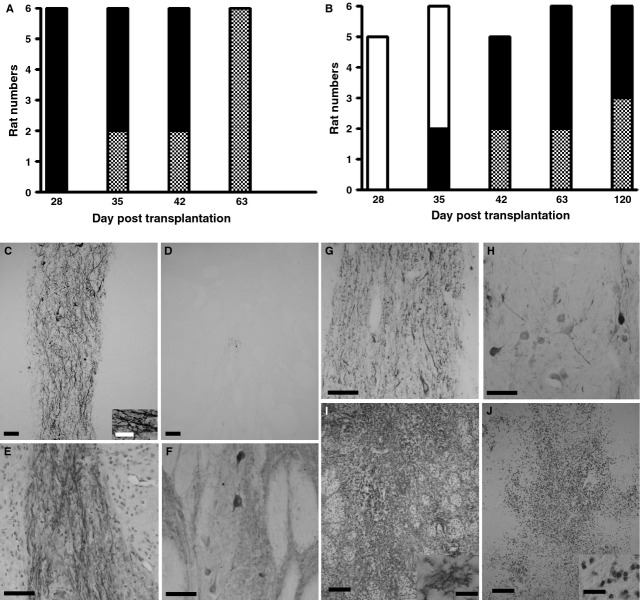
Immunohistological analyses of the xenotransplants. (**A**–**B**) Numbers of rats grafted with pNb (**A**) or pNb and MSC (**B**), displaying an NF70-negative healthy graft (white bar), an NF70-positive graft (black bar) or a rejected graft (grey bar). (**C**–**H**) Representative micrograph of healthy grafts following the transplantation of pNb alone (**C**) or in the presence of MSC (**D**–**H**). At D28, NF70 immunostaining was observed in the pNb graft (**C**), whereas no NF70^+^ cells were observed in the cografts (**D**). At later stages, such as D63 (**E** and **F**) and D120 (**G** and **H**), the cografts were strongly immunopositive for NF70 (**E** and **G**) and exhibited TH^+^ cells (**F** and **H**). (**I** and **J**) Representative micrograph of a pNb-MSC cograft undergoing rejection. The grafts were immunostained with OX42 (**I**) or R73 (**J**). Scale Bars: **C**–**H**, 50 μm; **I** and **J**, 150 μm; insert, 25 μm.

In the rats transplanted with pNb, all the transplants exhibited NF70^+^ cells at D28 (Fig. 2A and C). In contrast, no NF70^+^ cells were detected in any of the cotransplants at D28 (Fig. 2B and D). NF70^+^ cells were found in the cotransplants from D35, but only in two of six rats. This percentage increased with time, reaching 100% at D42 (Fig. 2B). Interestingly, TH^+^ cells were detected in all grafts as soon as they became immunopositive for NF70. In rats transplanted with pNb, TH^+^ cells were found in all transplants at D28. In rats cotransplanted with pNb and MSC, TH^+^ cells were detected at D35, but only in the two animals that were immunopositive for NF70. At D63 and D120, all of the healthy cotransplants exhibited clear NF70^+^ immunostaining and showed TH^+^ cells (Fig. 2E–H). To evaluate the potential migration of the MSC in the brain, the experiment was performed with GFP-MSC [17]. Thirty-five days after the transplantation of pNb and GFP-MSC into the rat striatum, fluorescent MSC were only observed within the graft. No migration of transplanted MSC was detected (data not shown).

### Characterization of the host cellular immune response

To characterize the cellular immune response, immunohistochemistry was performed with antibodies directed against activated microglial cells/macrophages (Ox42) and T cells (R73). As previously described, the transplantation of pNb into the striatum of adult immunocompetent rats induced a strong cellular response characterized by a massive infiltration of the graft by T cells and activated microglial cells between D28 and D42. In the rats cografted with MSC, infiltration of the graft by T cells (Fig. 2J) was never observed before D42 (Table 3), and at D120 50% of these animals exhibited healthy grafts without any T cells.

**Fig. 3:**
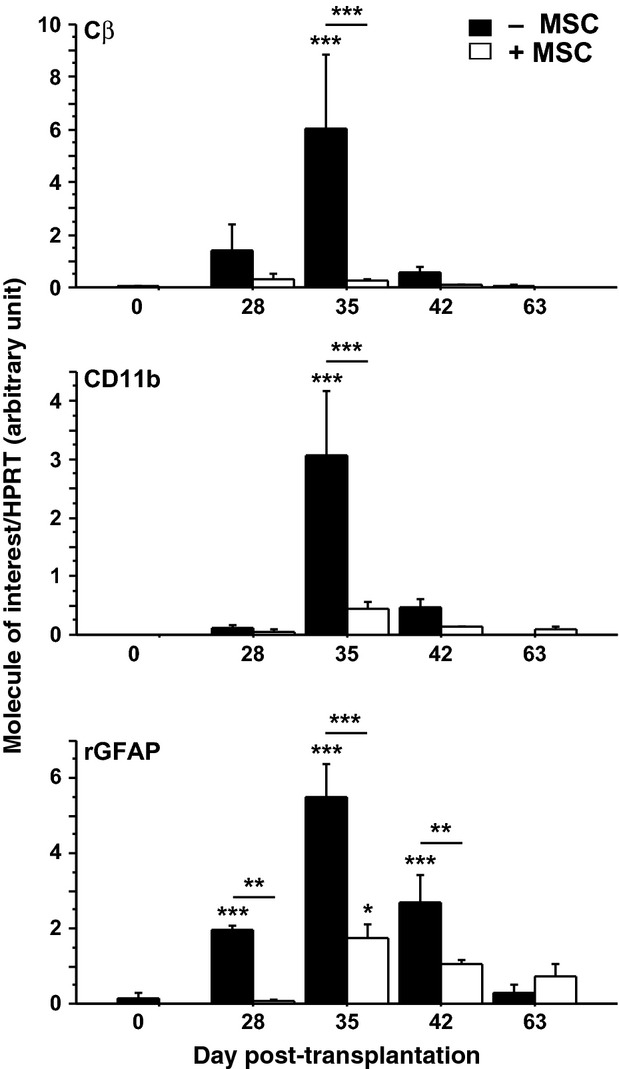
Relative expression of Cβ, CD11b and GFAP mRNAs in grafted animals. Total RNAs from striata grafted with pNb (−MSC), or pNb + MSC (+MSC), were collected at days 28, 35, 42, 63 (*n* = 7/8 per group per day) and submitted to qPCR. D0 corresponded to ungrafted animals (*n* = 6). Mean values ± SEM are presented (anova test, *P < 0.05; **P < 0.01; ***P < 0.001).

To evaluate the impact of MSC on inflammatory markers, the striata of rats transplanted with pNb or pNb-MSC were collected at 28, 35, 42 or 63 days post-transplantation for RNA analyses. Seven or eight animals were culled for each time-point in a given group. Striata from six sham-operated rats were used to determine the basal levels (control). We first analysed the expression of Cβ and CD11b as markers of T-cell infiltration and microglial cell activation respectively (Fig. 3). In rats transplanted with pNb, a sixfold and threefold increase in the levels of Cβ and CD11b mRNA was observed at D35, respectively, as compared with the basal levels in control rats. At later stages, such as D42 or D63, Cβ and CD11b mRNAs had returned to basal levels. In contrast, we did not observe significant increases in the levels of Cβ and CD11b in the rats transplanted with both pNb and MSC at any of the stages analysed. To evaluate a potential impact of MSC on host reactive astrocytes, we analysed the expression of rat GFAP transcripts. The results showed an up-regulation of GFAP mRNA at D28, preceding the peak of CD11b and Cβ. The levels of GFAP transcripts further increased, reaching a 5.4-fold increase at D35 and remaining significantly increased at D42. In the rats transplanted with both pNb and MSC, a significant increase in the levels of GFAP mRNA was observed only at D35, and this increase was only 1.7-fold.

**Fig. 4:**
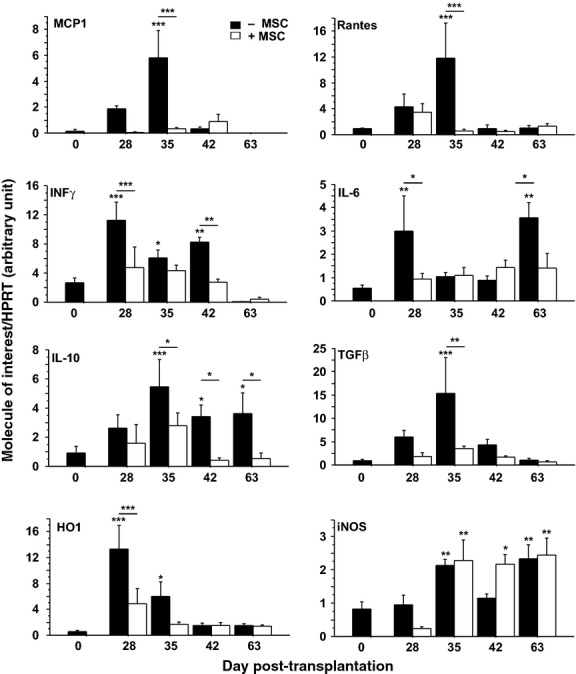
Relative transcriptional expression of chemokines, cytokines and cytoprotective enzymes in grafted animals. Total RNAs prepared from the striata of rats transplanted with pNb (−MSC), or pNb + MSC (+MSC), were collected at days 28, 35, 42, 63 and submitted to qPCR (*n* = 7/8 per group per day). D0 corresponds to untransplanted animals (*n* = 6). Mean values ± SEM are presented (anova test, *P < 0.05; **P < 0.01; ***P < 0.001).

**Fig. 5:**
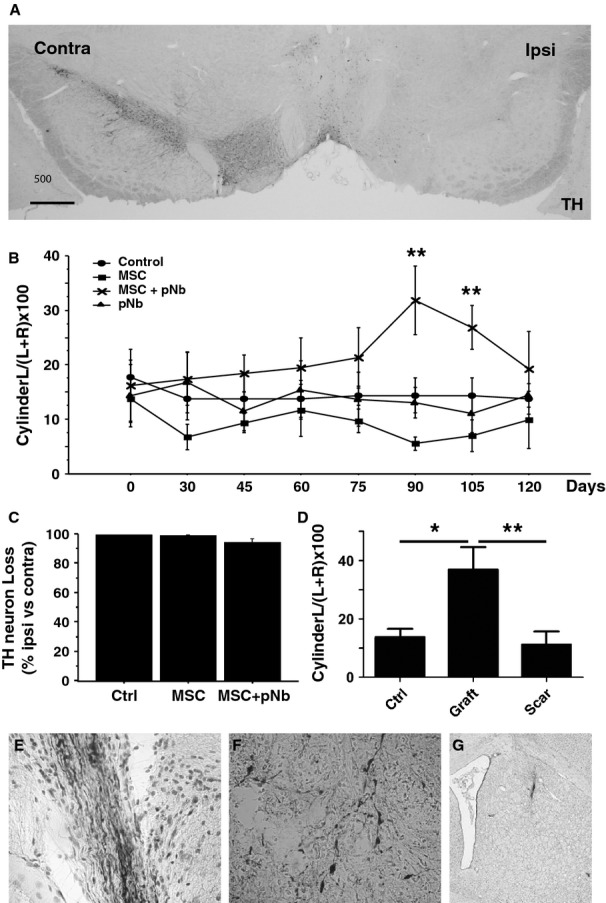
Functional recovery of 6-OHDA-lesioned rats. Rats unilaterally lesioned with 6-OHDA were transplanted with MSC, pNb, pNb+MSC, or vehicle (Control), and tested for motor recovery. (**A**) TH neuronal loss in the substantia nigra. TH immunostaining showing the loss of TH^+^ neurons in the substantia nigra ipsilateral (right) to the lesion. (**B**) Forelimb asymmetry was tested every 15 days with the cylinder test. Data are expressed as percentage of limb preference for the contralateral (left) forepaw. *n* = 8 per group, except *n* = 13 for the MSC + pNb group, mean value ± SEM (anova test, **P < 0.01). (**C**) The neuronal loss as a result of the 6-OHDA lesion was estimated by counting the numbers of TH^+^ cells in the substantia nigra of rats transplanted with MSC, with MSC+pNb, or untransplanted rats. Mean values ± SEM are presented. (**D**) Functional recovery according to the presence of transplant. Cylinder test scores at D120 were expressed according to the presence (Graft, *n* = 4) or absence (Scar, *n* = 9) of healthy graft in the rat striatum, and compared with the vehicle group (Crtl, *n* = 8). Mean values ± SEM are presented (one-way anova with Bonferroni post hoc test, *P < 0.05, **P < 0.01). (**E**–**G**) At D120, transplanted rats exhibited either a healthy graft with NF70^+^ (**E**, NF staining) and TH^+^ (**F**, TH staining) neurons or a scar (**G**, cresyl violet staining).

We next analysed the expression of chemokines and cytokines that might favour or inhibit the host immune response (Fig. 4). As reported in Figure 5, MCP1 and Rantes were significantly up-regulated at D35 in pNb-grafted animals, and the up-regulation of these two chemokines was correlated with the peak expression of Cβ and CD11b. We also found, at D28, an up-regulation of the pro-inflammatory cytokines, INFγ and IL-6, which preceded the up-regulation at D35 of anti-inflammatory cytokines such as IL-10 and TGF-β. The levels of interferon-γ (IFN-γ) remained high up to D42 and then strongly decreased, whereas IL-6 returned to its basal level before a second up-regulation at D63. A high level of IL-10 was also observed from D35 to D63, whereas TGF-β returned to its basal level as early as D42. Interestingly, we did not observe any of these regulations in the rats that received both pNb and MSC. The levels of MCP1 and RANTES (CCL5), as well as the levels of the four studied cytokines, were not significantly different from their basal levels in control animals at any of the time-points determined. To further characterize the immunomodulatory mechanism triggered by rat MSC in our experimental model, we analysed the expression of iNOS and HO1, two enzymes previously shown to be mediators of MSC function. As shown in Figure 4, the mRNAs of both of these enzymes were up-regulated in the striata of rats grafted with pNb, with the difference that HO1 expression increased between D28 and D42, whereas the level of iNOS increased between D35 and D63. Interestingly, in the presence of MSC, the up-regulation of HO1 was not observed, whereas the up-regulation of iNOS mRNA still occurred.

### Effect of cografting on the motor behaviour of 6-OHDA-lesioned rats

To evaluate the functional impact of a pNb-MSC cotransplant, the cells were transplanted in a rat model of Parkinson's disease. Rats lesioned by unilateral injection of 6-OHDA were selected on the basis of their response to the dopaminergic agonist, apomorphine. Post-mortem analysis confirmed a minimum of 90% loss of dopaminergic neurons in the substantia nigra pars compacta ipsilateral to the lesion (Fig. 5A and C). Lesioned rats were transplanted with pNb, or pNb + MSC and motor recovery was assessed every 15 days using the cylinder test. Sham-operated rats were used as controls, but 6-OHDA-lesioned rats were also transplanted with 1 × 10^5^ rMSC to evaluate a potential impact of this amount of MSC on the motor behaviour. As shown in Figure 5 B, no recovery was observed in the rats transplanted with MSC or pNb alone. In contrast, significant signs of motor improvement were observed at D90 and D105 in the group of rats that received both pNb and MSC. As the beneficial effect was no longer significant at D120, the rats were divided according to the presence or absence of transplant. At D120, the rats displayed either a scar (Fig. 5G) or a healthy transplant with NF70^+^ (Fig. 5E) and TH^+^ (Fig. 5F) cells. Intermediate stages were not observed. No activated microglial cells were observed in the control or pNb-MSC-transplanted rats. Inclusion of this parameter in the interpretation of the behavioural tests clearly showed significant recovery in rats displaying a healthy transplant (Fig. 5D). The rats that exhibited a healthy graft were those that presented the best performance in the cylinder test, whereas the performance of the rats that had rejected their graft was similar to that of the lesioned group that received vehicle (control). Motor recovery in 6-OHDA-lesioned rats was, therefore, correlated with the long-term survival of the pNb-MSC transplants.

## Discussion

Our present paper provides the first evidence that cotransplantation of syngeneic MSC with xenogeneic neuroblasts promotes the long-term survival of xenografted neurons and allows motor recovery in a rat model of Parkinson's disease. Behavioural improvement has previously been observed after the transplantation of pNb into the striatum of lesioned rats, but the animals were always treated with high doses of classic immunosuppressors [5,20,21]. Here, we show that pNb cotransplanted with rMSC can survive up to 120 days without systemic immunosuppression. This prolonged survival is most probably the result of an immunosuppressive activity, as the loss of xenografted neurons is because of a massive and sudden host immune response at 4–6 weeks [2,4]. This observation is supported by the description of MSC as potent immunosuppressors, acting at different levels of the immune response [13,14]. Human MSC alter the maturation of dendritic cells as well as their ability to present antigens to T cells, which is a key point of their activation [22]. They are also able to inhibit T-cell proliferation and to affect the differentiation of B cells into plasmocytes [23]. The MSC isolated from the bone marrow of Lewis 1A rats share similar immunosuppressive properties, as illustrated by their ability to inhibit the proliferation of *in vitro*-activated T cells and by the limited host immune response in pNb/MSCcografted rats. Indeed, numerous cellular and molecular events that are usually induced by the implantation of pNb in the rat brain are not observed or are strongly reduced in the presence of MSC. For instance, the accumulation of GFAP mRNA that precedes cell rejection in pNb-grafted rats is strongly reduced in cografted rats, suggesting an inhibition of astroglial activation. The absence of a significant increase in Cβ and CD11b levels at the analysed time-points probably reflects a direct or indirect inhibitory effect of MSC on T lymphocyte and microglial cell activation. *In vitro* analyses raise the possibility of an effect through the secretion of anti-inflammatory molecules such as TGF-β1 or IL10 [16]. However, *in vivo* analyses did not reveal up-regulation of such anti-inflammatory molecules. On the contrary, we observed a general abolition of pro- and anti-inflammatory signals. Indeed, MCP1, RANTES, IFN-γ, IL-6, IL-10, TGF-β and HO-1 remained at their basal levels in the striata of rats grafted with both pNb and MSC in contrast to their up-regulation at around D35 in the brains of pNb-grafted rats. Only one molecule was up-regulated in both groups: iNOS. This molecule might be implicated in the immunosuppressive effects of MSC through the production of NO. Indeed, Ren *et al*. revealed a major role of iNOS in the immunosuppressive properties of mouse MSC [24] and exacerbation of the EAE inflammatory response has been observed in mice deficient for iNOS [25]. The mechanism of action remains to be fully determined, but NO was reported to be a potent inhibitor of T-cell proliferation [26,27] and leucocyte adhesion on the endothelial cell layer [28]. A local overexpression of iNOS by MSC in the cograft might, therefore, favour the long-term survival of the intracerebral xenograft, but molecules other than NO are most probably implicated. Thus, further studies will be required to fully characterize the mechanisms by which MSC promote the long-term survival of xenografted pNb.

Neural stem/progenitor cells (NSPC) also display immunosuppressive properties and show prolonged survival in the brains of xenogenic hosts [19,29]. As they can generate neurons, these cells are considered to be a potential cell source for restorative strategies, but, in fact, very few pNSPC differentiate spontaneously into dopaminergic neurons *in vivo* [19]. We, thus, considered combining the ability of ventral mesencephalic neuroblasts to generate dopaminergic neurons and the immunosuppressive properties of MSC. The long-term survival of these cotransplants in the brains of xenogenic hosts and their functional effects in a rat model of Parkinson's disease suggest that the intracerebral implantation of 100,000 MSC with 300,000 pNb might be a good basis to develop new restorative strategies with high efficiency and low detrimental secondary effects. Characterization of the mechanisms by which locally implanted MSC contribute to motor recovery in immunocompetent rats should be of great help to optimize such strategies.

## References

[1] Krystkowiak P, Gaura V, Labalette M (2007). Alloimmunisation to donor antigens and immune rejection following foetal neural grafts to the brain in patients with Huntington's disease. PLoS ONE.

[2] Rémy S, Canova C, Daguin-Nerrière V (2001). Different mechanisms mediate the rejection of porcine neurons and endothelial cells transplanted into the rat brain. Xenotransplantation.

[3] Melchior B, Rémy S, Nerrière-Daguin V (2002). Temporal analysis of cytokine gene expression during infiltration of porcine neuronal grafts implanted into the rat brain. J Neurosci Res.

[4] Michel DC, Nerrière-Daguin V, Josien R (2006). Dendritic cell recruitment following xenografting of pig fetal mesencephalic cells into the rat brain. Exp Neurol.

[5] Galpern WR, Burns LH, Deacon TW (1996). Xenotransplantation of porcine fetal ventral mesencephalon in a rat model of Parkinson's disease: functional recovery and graft morphology. Exp Neurol.

[6] Larsson LC, Widner H (2000). Neural tissue xenografting. Scand J Immunol.

[7] Deacon T, Schumacher J, Dinsmore J (1997). Histological evidence of fetal pig neural cell survival after transplantation into a patient with Parkinson's disease. Nat Med.

[8] Fink JS, Schumacher JM, Ellias SL (2000). Porcine xenografts in Parkinson's disease and Huntington's disease patients: preliminary results. Cell Transplant.

[9] Schumacher JM, Ellias SA, Palmer EP (2000). Transplantation of embryonic porcine mesencephalic tissue in patients with PD. Neurology.

[10] Michel-Monigadon D, Nerrière-Daguin V, Lévèque X (2010). Minocycline promotes long-term survival of neuronal transplant in the brain by inhibiting late microglial activation and T-cell recruitment. Transplantation.

[11] Azizi SA, Stokes D, Augelli BJ (1998). Engraftment and migration of human bone marrow stromal cells implanted in the brains of albino rats–similarities to astrocyte grafts. Proc Natl Acad Sci USA.

[12] Rossignol J, Boyer C, Thinard R (2009). Mesenchymal stem cells induce a weak immune response in the rat striatum after allo or xenotransplantation. J Cell Mol Med.

[13] Uccelli A, Pistoia V, Moretta L (2007). Mesenchymal stem cells: a new strategy for immunosuppression?. Trends Immunol.

[14] Uccelli A, Moretta L, Pistoia V (2008). Mesenchymal stem cells in health and disease. Nat Rev Immunol.

[15] Zappia E, Casazza S, Pedemonte E (2005). Mesenchymal stem cells ameliorate experimental autoimmune encephalomyelitis inducing T-cell anergy. Blood.

[16] Lescaudron L, Boyer C, Bonnamain V (2012). Assessing the potential clinical utility of transplantations of neural and mesenchymal stem cells for treating neurodegenerative diseases. Methods Mol Biol.

[17] Remy S, Tesson L, Usal C (2010). New lines of GFP transgenic rats relevant for regenerative medicine and gene therapy. Transgenic Res.

[18] Schallert T, Fleming SM, Leasure JL (2000). CNS plasticity and assessment of forelimb sensorimotor outcome in unilateral rat models of stroke, cortical ablation, parkinsonism and spinal cord injury. Neuropharmacology.

[19] Michel-Monigadon D, Bonnamain V, Nerrière-Daguin V (2011). Trophic and immunoregulatory properties of neural precursor cells: benefit for intracerebral transplantation. Exp Neurol.

[20] Huffaker TK, Boss BD, Morgan AS (1989). Xenografting of fetal pig ventral mesencephalon corrects motor asymmetry in the rat model of Parkinson's disease. Exp Brain Res.

[21] Larsson LC, Czech KA, Brundin P (2000). Intrastriatal ventral mesencephalic xenografts of porcine tissue in rats: immune responses and functional effects. Cell Transplant.

[22] Maccario R, Moretta A, Cometa A (2005). Human mesenchymal stem cells and cyclosporin a exert a synergistic suppressive effect on *in vitro* activation of alloantigen-specific cytotoxic lymphocytes. Biol Blood Marrow Transplant.

[23] Corcione A, Benvenuto F, Ferretti E (2006). Human mesenchymal stem cells modulate B-cell functions. Blood.

[24] Ren G, Zhang L, Zhao X (2008). Mesenchymal stem cell-mediated immunosuppression occurs *via* concerted action of chemokines and nitric oxide. Cell Stem Cell.

[25] Fenyk-Melody JE, Garrison AE, Brunnert SR (1998). Experimental autoimmune encephalomyelitis is exacerbated in mice lacking the NOS2 gene. J Immunol.

[26] Albina JE, Abate JA, Henry WL (1991). Nitric oxide production is required for murine resident peritoneal macrophages to suppress mitogen-stimulated T cell proliferation. Role of IFN-gamma in the induction of the nitric oxide-synthesizing pathway. J Immunol.

[27] Krenger W, Falzarano G, Delmonte J (1996). Interferon-gamma suppresses T-cell proliferation to mitogen *via* the nitric oxide pathway during experimental acute graft-versus-host disease. Blood.

[28] Kubes P, Kurose I, Granger DN (1994). NO donors prevent integrin-induced leukocyte adhesion but not P-selectin-dependent rolling in postischemic venules. Am J Physiol.

[29] Bonnamain V, Mathieux E, Thinard R (2012). Expression of heme oxygenase-1 in neural stem/progenitor cells as a potential mechanism to evade host immune response. Stem Cells.

